# The outcomes of a person-centered, non-pharmacological intervention in reducing agitation in residents with dementia in Australian rural nursing homes

**DOI:** 10.1186/s12877-021-02151-8

**Published:** 2021-03-20

**Authors:** Vivian Isaac, Abraham Kuot, Mohammad Hamiduzzaman, Edward Strivens, Jennene Greenhill

**Affiliations:** 1 Rural and Remote Health South Australia, College of Medicine and Public Health, Flinders University, Po Box 852, Ral Ral Avenue, Renmark, SA 5341 Australia; 2 James Cook University & Clinical Director, Older Persons Health Services, Cairns and Hinterland Hospital and Health Service, Cairns, Queensland 4870 Australia

**Keywords:** Dementia, Person-centered care, Non-pharmacological intervention, Nursing homes, Rural Australia

## Abstract

**Background:**

There is limited best- practice evidence to address behavioral and psychiatric symptoms for those with dementia in Australian rural nursing homes. This study aims to evaluate the outcomes of a person-centered, non-pharmacological dementia care model, ‘Harmony in the Bush’, based on the Progressively Lowered Stress Threshold principles and person-centered music in rural Australia.

**Methods:**

A quasi-experimental (nonrandomized, pre-post) intervention study was conducted in five rural nursing homes in Queensland and South Australia. Seventy-four residents with dementia participated in this intervention study, which yielded a sample power of 80%. Eighty-seven staff completed the Caregiver Stress Inventory at pre-post four-weeks of intervention. Staff training workshops focused on the theory of the Progressively Lowered Stress Threshold principles and delivery of person-centered care plan with integrated music intervention. We used reported changes in agitation of the residents, measured using Cohen- Mansfield Agitation Inventory, and staff’s caregiving stress, using Caregivers Stress Inventory. This study adheres to the CONSORT guidelines.

**Results:**

Mean age of residents with dementia was 82.4 (7.7) years and 69% were females. The mean age of admission was 80.1(8.4) years. Baseline measures indicated that 32.7% had mild- severe pain and 30.5% reported mild-severe sadness. The results showed statistically significant decline in aggressive behaviors, physically non-aggressive behaviors, verbally agitated behavior and hiding and hoarding. There was similar reduction in staff stress in the domains of aggressive behaviors, inappropriate behaviors, resident safety, and resource deficiency.

**Conclusions:**

The Harmony in the Bush model is effective in reducing agitation among dementia residents with significant reduction in staff stress levels in nursing homes in rural Australia.

**Trial registration:**

Australian and New Zealand Clinical Trials Registry (ANZCTR) on 20/2/2018 (Registration No: ACTRN12618000263291p). https://www.anzctr.org.au/Trial/Registration/TrialReview.aspx?id=374458

## Background

In Australia, 60–70% of the people residing in nursing homes have dementia [1, 2] and about 70–90% of residents with dementia suffer from psychiatric or behavioral symptoms [1, 3]. These behaviors include agitation, mood dysregulation, and disturbed thoughts and perceptions, which pose a major challenge for the residents with dementia and nursing home staff [4, 5]. Behavioral and psychiatric symptoms are associated with diminished quality of life of the residents and increased difficulty associated with caregiving for the staff [5–10]. Agitation is often the most common challenging behavioral symptom being reported in 70% of dementia residents [11]. However, nursing home staff in rural Australia have poor understanding of appropriate methods of management and lack of adequate resources to manage behavioral and psychiatric symptoms, including agitation in dementia [12]. There is an overwhelming need to build best-practice evidence to address the residents’ behavioral problems, leading to improve patient outcomes and staff quality of life in rural nursing homes.

Evidence of non-pharmacological interventions is growing and have been recommended to be pursued at first instance, rather than pharmacological treatments, in the management of behavioral and psychiatric symptoms of residents with dementia. Non-pharmacological interventions are recommended to be person-centered to truly mitigate behavioral symptoms in residents with dementia [13]. Person-centered dementia care accentuates well-being and quality of life as regarded by the individual with dementia and their family members and creating an environment that considers the person’s past life and current status [14]. However, there is shortage of staff education and adequate training in rural nursing homes to develop the skills, knowledge and confidence in the use of evidence-based non-pharmacological interventions in person-centered care [12]. Staff turnover, challenges in recruiting and retaining skilled staff, lack of resources compared to metropolitan areas pose major challenges for rural nursing homes [9].

The Progressively Lowered Stress Threshold (PLST) is an established model that provides a framework for person-centered dementia care [6]. The model suggests that behaviors are in response to internal and external stimuli that may arise from the complex interactions between the residents and the nursing home environment. The model proposes three types of behaviours: normative, anxious and dysfunctional. Residents with dementia have reduced ability to experience stimuli, such as hunger, pain or transient noise and their stress level increases as an outcome [15]. Progressive neurological damage reduces the ability to receive and process information from the external environment gradually decreasing their stress threshold. When stressors are not attended, their lowered threshold to stressors will manifest as anxious behaviours and then eventually as dysfunctional behaviors. However, this model also assumes that all stress-related behaviors have an underlying cause and the caregivers can be trained to address the individuals needs and/or modify the environmental stressors to eliminate or minimize behavioral and psychiatric symptoms [7]. Environmental factors, for example, location, light, noise, and the surrounding atmosphere are associated with exaggeration of behavioral symptoms in dementia [16, 17]. Staff training, consideration of environmental adjustment, person-centered activities have been recommended as effective approaches for management of behavioral and psychiatric symptoms of dementia [18].

Music intervention is one of the non-pharmacological methods used to reduce behavioural and psychiatric symptoms in dementia. Person-centered music interventions are a promising non-pharmacological approach of dementia care in nursing homes [19]. Individualized preferred music that incorporates the cultural differences, personal memories, and individual taste has shown to improve attention, ability to access remote memory and emotions [20]. The music provides a stimulus that can be received and processed despite reduced processing capacity in dementia, thus stimulating memories and provides a positive soothing effect that can prevent stress level exceeding the stress threshold. However, a recent study did not show statistically significant decline in behavioral and psychiatric symptoms after 6 weeks of person-centered music intervention [21].

This intervention did not adopt PLST as a theoretical framework for staff education and employed music intervention as stand-alone intervention without a comprehensive person-centered care plan [22, 23]. PLST based person-centered care plan includes applying PLST based principles and strategies in day-day care, such as including tailored activities based on their ability and interest, address individual needs, reduce environmental stimuli and improve staff interaction. As such, a comprehensive PLST model that incorporates person-centered care plan and music intervention has not been tested previously, especially for residents with dementia living in rural nursing homes in Australia.

Harmony in the Bush is a co-designed dementia care model that integrates PLST based person- centered care plan and music (during rest time using MP3 players), for the development of a low stress, person-centered organizational culture. Person-centred music was adopted as a main component of the Harmony in the Bush study as it requires minimal training for staff and can be integrated into the PLST based care plan. This paper reports the 4-week outcome of the intervention on residents’ behavioral outcomes and staff stress in the nursing homes. It is hypothesized that this person-centered, non-pharmacological intervention will reduce agitative behaviours of residents with dementia with parallel reduction in caregiver stress in staff.

## Methods

### Research design and setting

Harmony in the Bush is a quasi-experimental study conducted in five rural nursing homes of Australia [South Australia (*n* = 2) and Queensland (*n* = 3)]. The nursing homes were purposely selected, and their managers were approached by the investigators and the former expressed interest to participate. To reflect on the diversity of the participating nursing homes, two were privately owned; one was a public funded (state-run center); and one was a not-for-profit facility. The fifth facility was a not-for- profit, partly funded by the government, Aboriginal Residential and Community Aged Care home.

The total number of residents living with dementia across the five participating aged care facilities in this this study is approximately 170–180. Allowing for 10% attrition, the investigators have estimated that 70–80 residents from this cohort can ensure a statistical power of 0.80. This estimation is based on a recent meta-analysis on effect of music interventions on agitation, which reports an overall effect size (Cohen’s d) of 0.61 calculated from 12 studies [24], and by adopting an alpha level of 0.05 and employing the algorithm [25]. This study adheres to the CONSORT guidelines.

### Participants

Participants included eligible residents with dementia and staff from the participating nursing homes. Resident participants were recruited following receipt of informed consents to participate in the study by their legal guardians/power of attorney appointees. In each of the four nursing homes, 15–20 residents were selected as potential participants; but in one aged care home, which has a total of 15 residents, all residents with dementia were approached to participate. As a result, a total of 77 residents with dementia were recruited as participants. Inclusion criteria for the residents were: (a) diagnosis of dementia within the Diagnostic and Statistical Manual of Mental Health Disorders 5 (DSM-5) [26]; (b) a Standardized Mini Mental Status Examination (SMMSE) score of less than 24 [10]; (c) being ambulant; and (d) displaying dysfunctional behaviors. The residents who could not meet the inclusion criteria or had a medical condition that affects normative behavioral patterns, for example history of schizophrenia were excluded. Nursing home staff, registered nurses, enrolled nurses, and care staff, were approached through the facility managers to participate in this research, resulting in 87 staff participants after voluntary consent. The staff participated in the PLST based educational program, observed, and recorded behaviours of the dementia participants and completed the Caregiver Stress Inventory (CSI).

### Data collection procedure and intervention

The study intervention included:

#### Baseline assessments (one week)

Demographic details and baseline clinical data were collected for each of the resident in the study. The demographic information included age, sex, education level and occupation. In this stage, each participant was assessed using the SMMSE or Kimberley Indigenous Cognitive Assessment (KICA-Cog); Pain Assessment in Advanced Dementia (PAINAD) scale; Cornell Scale for Depression in Dementia (CSDD) or Kimberley Indigenous Cognitive Assessment-Depression (KICA-Dep); Barthel Index of Activities of Daily Living (ADL); Apathy Evaluation Scale (AES); and Cohen-Mansfield Agitation Inventory (CMAI).

#### Education and development of person-centered care plan (three weeks)

The staff training workshops were conducted by an experience nurse educator. The training focused on symptoms associated with Alzheimer’s disease and related disorders, the theory and delivery of the PLST, person-centered dementia care and the use of the measurement instruments listed in stage 1 – Baseline assessment. The Harmony in the Bush intervention entailed the introduction of person-centered care plan based on the following principles of the PLST theory:

- Introduce consistent individualized routines to compensate for cognitive decline;
- Organize small group activities to eliminate overwhelming stimuli;
- Allow residents to set their own sleep/wake cycle to prevent fatigue;
- Plan activities based on past experiences and practices considering present cognitive and functional abilities; and
- Eliminate misleading stimuli that trigger illusions.

The expected learning outcome after completion of the training was that staff who attended training should be able to identify:

- Understanding of person-centered approach to dementia care and PLST principles
- One each of the cognitive, affective and conative losses associated with Alzheimer’s disease and related disorders
- Five non-cognitive symptoms associated with Alzheimer’s disease and related disorders; and
- Four stressors that may potentiate non-cognitive symptoms.
- Implement person-centered activities and music appropriate to each resident

All staff on duty were responsible for observing and recording the residents’ behavior on each shift. Two staff in each facility were appointed as change champions by senior managers and trained in the process to ensure sustainability of the research outcomes after the project completion.

#### Person-centered music

Individualized music forms the basis for the person-centered music intervention. Individualized music aims to identify specific music preferences including exact song/composition titles and performers. We used The Assessment of Personal Music Preference Protocol in the Evidence-Based Guideline of Individualized Music for Persons with Dementia [19] to assist in the process of music selection for each of the participating residents. The personalized music playlist was developed in consultation with the resident, the resident’s family member and the staff. The residents listened to the music for 20–30 min during rest periods and at other time requested by the residents where the situation was appropriate. Staff monitored the resident stimulation levels and adjusted music accordingly.

#### Intervention and post evaluation phase (four weeks)

**Fig. 1:**
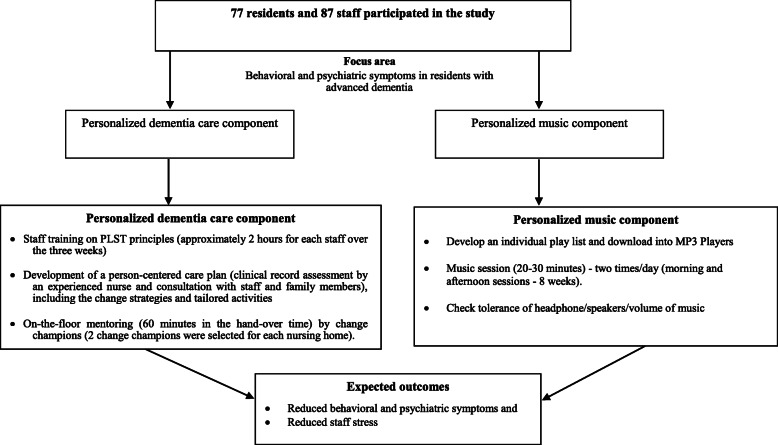
Harmony in the Bush personalized dementia care intervention

A person-centered care plan and personalized preferred music playlists were provided to participants based on the PLST principles [15] (Table 1; Fig. 1).

**Table 1:** Harmony in Bush Study: Intervention principles and strategies

Intervention Principles	Intervention Strategies
Maximize safe function by supporting losses in a person-centered manner	Activity based on their ability and preference (previous interests)
	To set their own sleep and wake-up cycle as much as possible
	Limit choices and serve food one after another and preferred menu
	Food and drink stall
	Familiar routine; Scheduled afternoon nap time
Provide unconditional positive regard	Polite language, address them with their names and treat them as mature adults
	Gentle touch
	Provide distraction instead of correction or argument
Use anxiety and avoidance to gauge activity and stimulation levels	Look for early signs of anxiety (toe tapping, fidgety) Provision of individualized music – preferred playlist
Teach caregivers to observe and listen to patients	Observe and listen to person’s needs Attend to their needs or stressors (internal or external)
Modify environment to support losses and enhance safety	Reduction of noise level in the environment (transient noise due to TV) Reduce outside noise (example mowing during rest time) Remove any hazards in the room and outside Reduce unnecessary lighting during sleep
Provide ongoing education, support and problem solving	On-going education; mentoring Building relationships among and between staff and residents

### Outcome measures

#### Baseline measures

- Standardized Mini Mental State Examination (SMMSE) [10], was used to measure cognitive function in residents. Kimberley Indigenous Cognitive Assessment (KICA-Cog) [27], was administered for the Indigenous participants. The cut-off levels were no cognitive impairment 24–30, mild cognitive impairment 19–23, moderate cognitive impairment 10–18 and severe cognitive impairment < 10.
- The Barthel Index of Activities of Daily Living (ADL) [28] (10-item), was administered to measure ADL of the residents. The total score ranges from 0 to 100.
- Pain Assessment in Advanced Dementia (PAINAD) scale was used to measure pain. PAINAD is a frequently used simple five-item observational tool with a range of 0 to 10 [29]. The five indicators were breathing, vocalization, facial expression, body language and consolability. The scores are categories as mild pain 1–3; moderate pain 4–6; severe pain 7–10.
- Cornell Scale for Depression in Dementia (CSDD) [30], was used to specifically assess signs and symptoms of a major depression in people with a dementia. The tool measures physical well-being, sleep, appetite and other vegetative symptoms. The Kimberley Indigenous Cognitive Assessment – Depression (KICA-Dep) [31], a culturally acceptable screening tool for depression among older Indigenous Australians living in remote areas, was alternatively administered for the Indigenous participants.

#### Baseline and post-intervention measures

- Cohen-Mansfield Agitation Inventory (CMAI) [32] was used in this study to assess level of agitation in residents with dementia. CMAI is a valid and reliable tool for assessing agitation in individuals living in residential aged care facilities. CMAI can be effectively administered by the nursing staff and direct care workers [33]. Cohen-Mansfield, Marx, and Rosenthal (1989) classified the manifestations of agitation into the following three syndromes: (a) aggressive behavior
  (e.g., hitting, kicking, cursing); (b) physical nonaggressive behavior (i.e., restlessness, pacing); and (c) verbally agitated behaviors (e.g., complaining, negativism, repetitious sentences). CMAI was administered consecutively for 5 days pre and post intervention. A maximum of three assessments per day consistent with staff shifts (7.00–15.00, 15.15–23.15, 23.30–7.15). An aggregate score that reflects the number of incidents per shift was calculated
- Caregiver Stress Inventory (CSI) [34] was used to measure staff stress levels. CSI is a 43-item, self-reporting tool developed to determine individual staff caregiver stress to behaviours that occur in residents with dementia. Staff stress is measured as an effect of nursing staff experience to the incidents and behaviours they manage in their day-day care of residents with dementia. The self-rated response was coded on a seven-point Likert-type scale (1 = not stressful, 7 = extremely stressful) based on current perceptions of stress. The tool consists of four subscales representing staff stress from aggressive behavior, inappropriate behavior, resident safety, and resource deficiency. The tool is valid for use with paid carers and informal carers and has been recently used in Australian residential care facilities [35]. The tools used in the study did not require license and were available for research purposes.

The paper reports the outcomes related to resident behavior and caregiver stress, while other elements of the Harmony in the Bush study such as sleep, and psychotropic medication use will be reported separately. We recently reported on the determinants of person-centered care in rural nursing homes using follow-up qualitative data from the Harmony in the Bush study [36].

### Data analyses

All analysis of pre- and post-test data was analyzed using SPSS IBM Version 23. Statistical tests were considered significant at *p* < 0.05. Descriptive data was checked for outliers and assumptions for parametric analyses. Paired-t test was used to analyze the difference between baseline and 4 weeks follow-up of CMAI and CSI scores. Cross tabulation and chi-square tests were used to analyze the factors associated with caregiver stress. Repeated measures ANOVA were used to understand difference in baseline and follow-up assessments to understand the changes in behavioral and psychological symptoms in residents after adjustments for age and sex. Similarly, repeated measures ANOVA were used to analyze changes in staff stress in caregiving measured using CSI adjusted for age and sex.

## Results

Data was available for 74 residents with dementia who took part in the intervention. Their demographic characteristics and baseline measures for the participants are described in Table 2. The mean age of the participants was 82.4 (7.7) years, and 68.9% were females. The mean age of admission of the residents was 80.1(8.4) years. The mean SMMSE score was 9.21 (6.7); 45.9% has SMMSE score less than 10. The PAINAD scale indicated that 23.0% had mild pain and 9.5% with moderate to severe pain at baseline. On the Cornell Scale for Depression in Dementia (CSDD), a significant 68.9% reported anxiety symptoms, 40.5% had mild-severe sadness, 41.9% reported mild- severe irritability and 46.0% exhibited mild-severe agitation. Barthel Index score of 58.4 (24.0) for Activities of Daily Living (ADL) was measured in residents with dementia at the baseline.

**Table 2:** Demographic characteristics of the dementia residents at baseline

	N (%)
Sex	
Female	51 (68.9)
Male	23 (31.1)
Age (mean, SD)	82.4 (7.7)
Education	
None	23 (31.1)
Primary school	15 (20.3)
High school	19 (25.7)
University degree	6 (8.1)
Previous occupation	
Unemployed	11 (14.9)
Factory or farm worker	44 (59.5)
Administration or officer	11 (14.9)
University or government	4 (5.4)
Marital status	
Married	30 (40.5)
Divorced	6 (8.1)
Widowed	33 (44.6)
Age at admission (mean, SD)	80.1 (8.4)
Duration of stay	
0–2 years	42 (56.8)
2–5 years	19 (25.7)
> 5 years	13 (17.6)
SMMSE (mean, SD)	9.21 (6.7)
Cognitive impairment, n (%)	
Severe	34 (45.9)
Moderate	27 (36.5)
Mild	6 (8.1)
Pain	
None	43 (58.1)
Mild pain	17 (23.0)
Moderate pain	5 (6.8)
Server pain	2 (2.7)
Barthel Index (mean, SD)	58.4 (24.0)
Cornell Depression Scale	
Anxiety	
Unable to evaluate	3 (4.1)
Absent	16 (21.6)
Mild or intermittent	35 (47.3)
Severe	16 (21.6)
Sadness	
Unable to evaluate	2 (2.7)
Absent	37 (50.0)
Mild or intermittent	22 (29.7)
Severe	8 (10.8)
Irritability	
Unable to evaluate	4 (5.4)
Absent	32 (43.2)
Mild or intermittent	23 (31.1)
Severe	8 (10.8)
Agitation	
Unable to evaluate	3 (4.1)
Absent	32 (43.2)
Mild or intermittent	25 (33.8)
Severe	9 (12.2)

Percentage does not add up to 100% due to non-response

### Effectiveness of the intervention in reducing agitation

Table 3 illustrates the change in behaviors in residents with dementia, pre- and -post- intervention. The common symptoms at baseline were pacing, aimless wandering (86.5%); general restlessness (74.3%); trying to get to a different place (63.5%); repetitive sentences (60.8%); cursing and verbal aggression (55.4%). Paired analyses between baseline and post-intervention observed significant decline in behavioral and psychological symptoms of dementia across domains, in resident participants (Table 4). The mean scores total CMAI declined from 3.05/shift to 1.35/shift after 4 weeks (paired t = 8.5, < 0.001). Analyses using repeated measure ANOVA indicated the decline in scores was significant after controlling for age and sex (F = 6.3. *p* = 0.015).

**Table 3:** Prevalence of behavioral and psychological symptoms in residents with dementia at baseline and 4 weeks

Domain	Symptoms	Baseline n (%)	4 weeks n (%)
Aggressive behavior	Hitting	12 (16.2)	16 (21.6)
	Kicking	6 (8.1)	10 (13.5)
	Grabbing onto people	25 (33.8)	22 (29.7)
	Pushing	20 (27.0)	16 (21.6)
	Throwing things	7 (9.5)	13 (17.6)
	Biting	3 (4.1)	8 (10.8)
	Scratching	6 (8.1)	10 (13.5)
	Spitting	12 (16.2)	12 (16.2)
	Hurt self or others	8 (10.8)	9 (12.2)
	Tearing things or destroying property	13 (17.6)	11 (14.9)
	Screaming	17 (23.0)	17 (23.0)
	Cursing and verbal aggression	41 (55.4)	28 (37.8)
Physically non-aggressive behavior			
	Paces, aimless wandering	64 (86.5)	49 (66.2)
	Inappropriate dress or disrobing	33 (44.6)	23 (31.1)
	Trying to get to a different place	47 (63.5)	21 (28.4)
	Handling things inappropriately	36 (48.6)	16 (21.6)
	Performing repetitious mannerisms	32 (43.2)	23 (31.1)
	General restlessness	55 (74.3)	40 (54.1)
Verbally agitated behavior			
	Repetitive sentences	45 (60.8)	20 (27.0)
	Complaining	30 (40.5)	15 (20.3)
	Negativism	33 (44.6)	18 (24.3)
	Constant unwarranted request for attention or help	31 (41.9)	15 (20.3)
Hiding and Hoarding	Hiding	20 (27.0)	11 (14.9)
	Hoarding	23 (31.1)	13 (17.6)

**Table 4:** Effectiveness of intervention on changed behaviors of residents with dementia

	Baseline	4-weeeks	Paired t-test	*Adjusted for age and sex
Aggressive behavior	0.43 (0.65)	0.21 (0.29)	3.6 (66), 0.001	F = 9.8, *p* = 0.003
Physically non-aggressive behavior	1.88 (1.71)	0.94 (1.19)	6.5 (66), < 0.001	F = 30.6, *p* < 0.001
Verbally agitated behavior	0.55 (0.69)	0.15 (0.34)	5.7 (66), < 0.001	F = 9.9, *p* = 0.002
Hiding and Hoarding	0.10 (0.19)	0.04 (0.19)	2.3 (66), < 0.001	F = 2.2, *p* = 0.15
Total CMAI	3.05 (2.54)	1.35 (1.50)	8.5 (66), < 0.001	F = 6.3. *p =* 0.015

### Effectiveness of the intervention in reducing staff stress in caregiving

Of the nursing home staff involved in the Harmony in the Bush intervention, 87 completed the Caregiver Stress Inventory questionnaires at baseline (*n* = 87) and 4 weeks post-intervention (*n* = 58). About 39 completed both baseline and follow-up measures. The changing work shifts, and staff turnover reduced the response rate for follow-up assessment. The mean age of staff was 44.9 (13.5) years, 84.1% were female. Among the staff who completed the CSI, 69.3% were personal carers, 18.2% were registered nurses, and 11.4% were enrolled nurses. More than one-third of the staff had country of origin other than Australia and the majority (59.8%) had work experience of more than 6 years in nursing homes.

The Caregiver Stress Inventory scores are presented in four domains i.e., aggressive behavior, inappropriate behavior, resident safety, and resource deficiency. The mean score of Total CSI was 3.6 (0.92) at baseline. Table 5 explains the factors associated with baseline Caregiver Stress Inventory scores. Female staff reported higher mean scores than males regarding stress related to inappropriate behavior (t = − 2.3, *p* = 0.02). The other significant difference was that staff whose country of birth was not Australia reported high stress scores in the domains of aggressive behaviors (t = − 1.8, *p* = 0.07) and resource deficiency (t = − 2.3, *p =* 0.02).

**Table 5:** Factors associated with caregiver stress at baseline

	Aggressive behavior		Inappropriate behavior		Resident safety		Resource deficiency		Total CSI	
	Mean (SD)	F/t (*p* value)	Mean (SD)	F/t (p value)	Mean (SD)	F/t (p value)	Mean (SD)	F/t (p value)	Mean (SD)	
Age										
< 35	4.5 (1.2)	0.3 (0.74)	3.4 (0.9)	0.4 (0.63)	4.2 (1.2)	0.1 (0.88)	3.9 (1.3)	1.7 (0.18)	3.9 (1.0)	0.7 (0.47)
35–55	4.2 (1.2)		3.1 (0.9)		4.1 (1.1)		3.4 (1.1)		3.6 (0.9)	
> 55	4.4 (0.9)		3.3 (0.9)		4.3 (0.8)		3.4 (1.0)		3.7 (0.8)	
Sex										
Male	4.0 (1.3)	−1.1 (0.25)	2.7 (0.7)	−2.3 (0.02)	3.9 (1.1)	−1.2 (0.21)	3.4 (1.3)	−0.5 (0.5)	3.2 (0.8)	−2.0 (0.05)
Female	4.4 (1.1)		3.4 (0.9)		4.3 (1.0)		3.6 (1.1)		3.8 (0.9)	
Position										
Personal Carer	4.2 (1.1)	−1.1 (0.26)	3.2 (0.9)	−1.0 (0.30)	4.2 (1.1)	−0.3 (0.76)	3.5 (1.2)	−0.2 (0.85)	3.6 (0.9)	−1.0 (0.31)
Registered/Enrolled nurse	4.5 (1.1)		3.4 (0.9)		4.3 (0.9)		3.6 (1.0)		3.8 (0.9)	
Country of birth										
Australia	4.1 (1.1)	−1.8 (0.07)	3.1 (1.0)	−1.1 (0.23)	4.1 (1.2)	−1.4 (0.15)	3.3 (1.2)	−2.3 (0.02)	3.5 (0.7)	−1.8 (0.07)
Others	4.6 (1.0)		3.4 (0.8)		4.4 (0.9)		3.9 (1.0)		3.9 (0.7)	
Work experience										
< 5 year	4.2 (1.1)	0.3 (0.5)	3.2 (0.9)	0.3 (0.56)	4.2 (1.1)	0.1 (0.73)	3.5 (1.3)	0.01 (0.89)	3.6 (0.9)	0.08 (0.77)
6–10 years	4.4 (1.1)		3.3 (0.9)		4.2 (1.0)		3.6 (1.1)		3.7 (0.9)	
> 10 years	4.3 (1.1)		3.2 (0.9)		4.2 (1.1)		3.5 (1.2)		3.7 (0.9)	

Table 6 explains the change in CSI score between baseline and after 4 weeks. The total CSI reduced from 3.6 (0.9) to 3.1 (1.1) between the two time points. Paired analyses of 39 staff showed consistent decline in staff stress in all subscales. Repeated measures ANOVA indicated that the decline in caregiver stress was significant after controlling for age and sex (F = 4.3, *p* = 0.05).

**Table 6:** Effectiveness of intervention on stress associated caregiving at baseline and 4 weeks

	Baseline	4-weeks	Paired t-test	*Adjusted for age and sex
Aggressive behavior	4.3 (1.1)	3.5 (1.4)	4.3 (38), 0.001	F = 6.8, *p =* 0.01
Inappropriate behavior	3.2 (0.9)	2.7 (1.1)	3.0 (37), 0.005	F = 1.6, *p* = 0.20
Resident safety	4.2 (1.0)	3.5 (1.2)	4.0 (37), < 0.001	F = 4.3, *p* = 0.04
Resource deficiency	3.6 (1.2)	3.0 (1.1)	2.9 (38), 0.006	F = 2.3, *p* = 0.13
Total CSI	3.6 (0.9)	3.1 (1.1)	3.8 (36), 0.001	F = 4.3. *p =* 0.05

## Discussion

The present study provides promising evidence that ‘Harmony in the Bush’ model results in the reduction of behavioral symptoms in residents and reduces staff stress in caregiving. We found statistically significant decline in aggressive behaviors, physically non-aggressive behaviors, verbally agitated behavior and hiding and hoarding behaviors in the study sample after 4 weeks of intervention in persons with dementia. Similarly, there was reduction in staff stress in the domains of aggressive behaviors, inappropriate behaviors, resident safety, and resource deficiency measured using Caregiver Stress Inventory tool. Thus, a person-centered, non- pharmacological intervention using PLST framework was effective in rural nursing home settings.

Our study results were consistent with a previous study, which found staff education and care based on the PLST model was clinically effective in reduction of behavior problems of care recipients, decreased caregiver burden and depression of family members, and in increasing their quality of life [37]. Similarly, Huang et al. [38] showed reduction in physically non-aggressive behavior, verbally aggressive and non-aggressive behavior subscales as well as the overall CMAI decreased significantly after 3 weeks of PLST intervention and improved self-efficacy of family caregivers. However, we found that some behaviours increased at 4 weeks such as hitting, kicking, throwing things, biting, hurting self or others. We postulate that the close contact and increased interaction between staff and resident with dementia during the intervention period compared to baseline may have resulted in these behaviours being recorded by the staff. Our study adds to the knowledge base of PLST based interventions and illustrates the effectiveness of the PLST framework for a person-centered care intervention in reducing behavioral and psychiatric symptoms in residents with dementia in rural Australian nursing home.

Our study also illustrates that implementation of individualised music as part of routine care by nursing and direct care workers provide a great avenue for person-centered care in rural nursing homes. While there is growing evidence of the positive impact of music in dementia care [24]; most are therapist-led music interventions, which are difficult to sustain in low-resourced rural nursing homes. Evidence that music therapy served advantages over non-therapist led listening is minimal [39, 40]. Several studies have found potential benefit of person-centered music intervention in dementia care. For example, listening to favorite music was effective in reducing agitation [41]. Other studies have revealed the use of pre-recorded personalized music can be effective in reducing anxiety, depression, pain and emotional well-being including quality of life [22, 39, 42]. Many of these studies have been criticized on methodological limitation and were suggested as low-quality evidence [43]. Our intervention includes a person-centered care plan with tailored activities and creating a low-stress, person-centered environment for listening to music. For example, environmental changes include reduce television noise, external noise for example mowing during rest time, adjustment of lighting during sleep. The benefit of utilizing a pre-recorded music playlist intervention as an alternative to formal music therapy is that the pre- recorded individualized music playlist is easy to access and can be conducted by nursing and direct care workers as part of their daily care regime.

Best practices to improve dementia care in nursing homes are difficult to establish and comply with factors intrinsic to the resident and complexity of care relating to dementia. Rural nursing home staff are highly stressed due to factors including long hours and heavy workload in managing the complex needs of the residents [9]. Further, higher prevalence of behavioral and psychiatric symptoms in residents with dementia correlates with higher stress in the caregivers [37, 44]. Poor knowledge of behavioral and psychiatric symptoms increases staff stress in caregiving and affects the quality of care [12]. Inadequate training and knowledge can lead to dementia residents being subjected to stigmatization and unnecessary use of restraint [45]. Our study included training for staff on behavior and psychiatric symptoms in dementia and the PLST framework for a person-centered care plan and music intervention. Studies have found that positive attitudes towards dementia and person-centered care correlated with job satisfaction [45, 46]. We anticipate staff training in person- centered care will increase quality of care to the residents, job satisfaction and retention in rural nursing homes.

The present study explains a best practice model for dementia care in Australian rural nursing homes with a good sample of 77 participants which yielded sample power of 80%. The quasi-experimental pre-post design was appropriate and feasible in rural nursing homes and the measurement tools were reliable and validated. However, there are some inherent limitations in the study. The lack of a control group prevented comparison with treatment as normal practice. The behaviours were recorded by the staff in each shift, which were collated by the research nurse/investigator and later analysed by the investigators. While the training; on-floor mentoring and frequent audit by the research nurse to ensure study fidelity, we also acknowledge there are potential variations in the exposure of intervention for each participant based on their interest and behaviours. Additionally, we were not able to disassociate the effect related to environmental modification, attitude change or music intervention on patient and carer outcomes. We also acknowledge the changing work shifts, and staff turnover reduced the response rate for follow-up assessment of staff stress.

## Conclusion

Harmony in the Bush model facilitates and provides a structure for staff education in person-centered practice to reduce the impact of behavioral and psychiatric symptoms of dementia among residents and improve staff wellbeing in rural Australia. Future investigations of this Harmony in the Bush model needs to employ controlled trial approach to determine the reliability and cost-effectiveness of the model.

## Data Availability

Data cannot be shared publicly because of the terms and conditions contained within the ethics permissions granted for this study from Southern Adelaide Clinical Research Ethics Committee, Australia and consented by participants.

## Abbreviations

PLST: Progressively Lowered Stress Threshold
SMMSE: Standardized Mini Mental State Examination
CMAI: Cohen-Mansfield Agitation Inventory
CSI: Caregiver Stress Inventory
PAINAD: Pain Assessment in Advanced Dementia (PAINAD)
ADL: Activities of Daily Living
CSDD: Cornell Scale for Depression in Dementia
